# A Nucleolus-Predominant *piggyBac* Transposase, NP-mPB, Mediates Elevated Transposition Efficiency in Mammalian Cells

**DOI:** 10.1371/journal.pone.0089396

**Published:** 2014-02-24

**Authors:** Jin-Bon Hong, Fu-Ju Chou, Amy T. Ku, Hsiang-Hsuan Fan, Tung-Lung Lee, Yung-Hsin Huang, Tsung-Lin Yang, I-Chang Su, I-Shing Yu, Shu-Wha Lin, Chung-Liang Chien, Hong-Nerng Ho, You-Tzung Chen

**Affiliations:** 1 Graduate Institute of Medical Genomics and Proteomics, National Taiwan University College of Medicine, Taipei, Taiwan; 2 Department of Dermatology, National Taiwan University Hospital and College of Medicine, Taipei, Taiwan; 3 Department of Otolaryngology, National Taiwan University Hospital and College of Medicine, Taipei, Taiwan; 4 Research Center for Developmental Biology and Regenerative Medicine, National Taiwan University, Taipei, Taiwan; 5 Graduate Institute of Clinical Medicine, National Taiwan University College of Medicine, Taipei, Taiwan; 6 Transgenic Mouse Model Core Facility of the National Research Program for Genomic Medicine, National Taiwan University College of Medicine, Taipei, Taiwan; 7 Laboratory Animal Center, National Taiwan University College of Medicine, Taipei, Taiwan; 8 Department of Clinical Laboratory Sciences and Medical Biotechnology, National Taiwan University College of Medicine, Taipei, Taiwan; 9 Department of Laboratory Medicine, National Taiwan University Hospital, Taipei, Taiwan; 10 Stem Cell Core Laboratory, National Taiwan University Center of Genomic Medicine, National Taiwan University, Taipei, Taiwan; 11 Graduate Institute of Anatomy and Cell Biology, National Taiwan University College of Medicine, Taipei, Taiwan; 12 Division of Reproductive Endocrinology and Infertility, Department of Obstetrics and Gynecology, National Taiwan University Hospital and College of Medicine, Taipei, Taiwan; 13 Genome and Systems Biology Program, National Taiwan University, Taipei, Taiwan; Institute of Hydrobiology, Chinese Academy of Sciences, China

## Abstract

*PiggyBac* is a prevalent transposon system used to deliver transgenes and functionally explore the mammalian untouched genomic territory. The important features of *piggyBac* transposon are the relatively low insertion site preference and the ability of seamless removal from genome, which allow its potential uses in functional genomics and regenerative medicine. Efforts to increase its transposition efficiency in mammals were made through engineering the corresponding transposase (PBase) codon usage to enhance its expression level and through screening for mutant PBase variants with increased enzyme activity. To improve the safety for its potential use in regenerative medicine applications, site-specific transposition was achieved by using engineered zinc finger- and Gal4-fused PBases. An excision-prone PBase variant has also been successfully developed. Here we describe the construction of a nucleolus-predominant PBase, NP-mPB, by adding a nucleolus-predominant (NP) signal peptide from HIV-1 TAT protein to a mammalian codon-optimized PBase (mPB). Although there is a predominant fraction of the NP-mPB-tGFP fusion proteins concentrated in the nucleoli, an insertion site preference toward nucleolar organizer regions is not detected. Instead a 3–4 fold increase in *piggyBac* transposition efficiency is reproducibly observed in mouse and human cells.

## Introduction

First cloned from the cabbage looper moth *Trichoplusia ni*, *piggyBac* is a class II DNA transposon that mobilizes DNA segments in a “cut-and paste” manner [1]. The *piggyBac* transposase (PBase) system has been widely applied as a genomic manipulation tool to various mammalian cell lines and model organisms, such as plants, cattle, pig, mouse, rat, rabbit, chicken, worms, fly, mosquito, planarian, yeast, protists, and several non-model insects [2]–[23]. Major features of the *piggyBac* system include a high transposition efficiency in different species, large cargo size, seamless removal, and relatively low insertion site preference (other than the conserved TTAA integration sequence) [3], [19], [24]–[26]. Owing to these features, the system has been used in many functional genomics studies, with particular utility for genes that are difficult to reach by other types of insertional mutagenesis vectors (*e.g.*, retroviruses and other transposons). Recent mutagenetic studies with the *piggyBac* system have been performed in mammalian gametes, embryonic stem (ES) cells, somatic cells, and cancer cell lines [7], [27]–[41].

The *piggyBac* system is also a candidate tool for regenerative medicine applications [42]–[44]. For induced pluripotent stem cell research, *piggyBac* can carry reprograming factors that enter and exit the genome without changing any nucleotides [45]–[48]. The *piggyBac* system has been applied to *in vitro* gene correction research designs in stem cells, to aid in the complete removal of a *piggyBac* inverted terminal repeat (ITR)-flanked drug selectable marker sequence from an exon without changing an encoded amino acid after genomic manipulations [49].

The transpositional function of mammalian codon-optimized PBase (mPB) can be maintained after mPB is fused with other proteins [34], [50]. For example, Cadinanos and Bradley fused PBase with a mutant estrogen receptor variant. Through this fusion, PBase was able to access the nucleus and mediate transposition, but only upon treatment with a steroid compound (tamoxifen) [50]. In another study, the AAV Rep-PBase fusion protein exhibited enriched capability for transposon insertion at Rep recognition sequences in the human genome [51]. Wilson *et al.* fused a site-specific synthetic zinc-finger DNA-binding domain (ZNF) to the N-terminus of *mPB*. The chimeric ZNF-mPB transposase exhibited a higher rate of site-directed genomic integration than the native mPB [52], [53]. Owens *et al.* fused the Gal4 DNA-binding domain (DBD) to mPB, and the chimeric Gal4-mPB facilitated transposon integration near artificially introduced upstream activating sequences [54].Transcription activator-like effector (TALE) is a new DNA-binding protein derived from the *Xanthomonas sp* [55], [56]. TALEs contain 34-amino acid tandem-repeat modules, which can be rearranged to target new DNA sequences [57], [58]. A TALE-linked PBase was demonstrated to direct transposition to a target region [59]. Protein function can be executed more efficiently by targeting the protein to a specific subcellular compartment. For example, a nuclear localization signal is used to direct recombinases and transcription factors to the nucleus, thereby increasing their access to DNA and other cofactors. Protein transduction domains (PTDs) are used to transfer cargo proteins efficiently across the biological membrane [11], [60], [61].

Here, we report a fused mPB with an 11-amino acid peptide from the HIV-1 transactivator of transcription (TAT) protein, which contains a short stretch of signal peptide (GRKKR) that is essential to direct TAT predominantly to the nucleolar compartment [62], [63]. This fusion transposase is designated as “nucleolus-predominant (NP)-mPB”. The nucleolus is a three-layer structure composed of nucleolar factors, such as fibrillarin, and chromosomal regions consisting of tandem-repeat sequences of ribosomal genes (rRNAs), called nucleolar organizer regions (NORs). In human, NORs include the 5S, 8S, 18S, and 28S rRNA gene clusters on the short arms of chromosomes 13, 14, 15, 21, and 22. Because there are hundreds of rRNA repeats in the genome, a transposon that jumps into an NOR is unlikely to disrupt a unique, essential gene and cause a phenotypic consequence. Therefore, we chose to target our PBase to the proximity of NORs by adding an NP signal peptide to its N-terminus. Although the subcellular distribution of this modified NP-mPB was concentrated (but not limited to) nucleoli, transposon insertion site preference towards NORs was not observed. Interestingly, both mouse and human cell lines demonstrated a reproducible 3- to 4-fold increase in transposition efficiency compared to mPB.

## Materials and Methods

### Transposase Vector Construction

The *pTriEX-HTNC* plasmid contained a fusion open reading frame (ORF) encoding six histidines, a stretch of the HIV-1 TAT sequence (including the NP signal peptide, GRKKR), and the phage P1 cyclization recombinase (Cre)-encoding sequence [60]. The NP signal peptide (underlined) was encoded in the following nucleotide sequence for the PTD: 5′-GGTCGCAAGA AACGTCGCCA ACGTCGCCGT CCGCCTGCA-3′.

To generate the *pTriEx-NP-mPB* transposase construct, the coding sequence of the mPB was cloned into the *pTriEX-HTNC* plasmid by replacing the Cre-encoding sequence restricted by *Spe*I and *Xho*I (New England Biolabs Inc., Ipswich, MA, USA). The *pTriEX-mPB* vector was constructed by removing the NP-encoding sequence from *pTriEX-NP-mPB*. The *pTriEx-mPB-tGFP* and *pTriEx-NP-mPB-tGFP* plasmids encode fusion ORFs consisting of the *PBase* variants and a *turboGFP* (*tGFP)* sequence from a *pGIPZ* plasmid (Thermo Fisher Scientific Inc., Waltham, MA, USA). The *pTriEx-mPB-2A-eGFP* and *pTriEx-NP-mPB-2A-eGFP* plasmids carried ORFs linking the *PBase* variants to *eGFP* by a self-cleaving T2A peptide-encoding sequence (5′- GAAGGACGAG GATCACTACT AACATGTGGA GACGTAGAAG AGAACCCAGG ACCT-3′).

The transposon construct used to test transposition efficiency was previously described [16]. Briefly, the *pXL-T3-Neo-UGm-cHS4X* (*UGm*) plasmid consisted of a human ubiquitin C promoter-driven bicolor fluorescent protein-labeling cassette, *H2B-EGFP-2A-mCherry-GPI* (Gm), flanked by two copies of chicken beta-globin insulators (2× Ins). A *PGK-neo-bpA* (Neo^r^) drug-selectable cassette was inserted between the *piggyBac* inverted repeats.

### Cell Culture

Mouse AB1 ES cells (kindly provided by Dr. Allan Bradley) [64], [65] were cultured in M15 medium (Dulbecco’s modified Eagle’s medium [DMEM] plus 15% fetal calf serum [FCS]) and maintained on irradiated SNLPb 76/7 feeders. Human H9 ES cells (National Stem Cell Bank, WiCell Research Institute, Madison, WI, USA) were maintained on irradiated feeders in human ES cell culture medium, consisting of 20% Knockout Serum Replacement (Invitrogen, Madison, WI, USA), 1 mM L-glutamine (Invitrogen), 0.1 mM β-mercaptoethanol (Sigma, St. Louis, MO, USA), 1% nonessential amino acids, and 40 ng/mL recombinant zebrafish basic fibroblast growth factor in DMEM-F12 (Invitrogen). Human ES cells were incubated at 37°C in 5% CO_2_ and passaged every 5 to 7 days with collagenase IV (Invitrogen). Hela cells (ATCC CCL-2) and HEK293T cells (ATCC CRL-11268) were cultured in DMEM containing 10% heat-inactivated FCS and 2 mM L-glutamine. Hela cell cultures at 80% confluence were passaged with trypsin.

### Measurement of Transposition Efficiency

Procedures for electroporation and drug selection for the mouse [65] and human [16] ES cells were performed according to previous studies. Briefly, to determine the difference in transposition efficiency between mPB and NP-mPB transposase, 1×10^7^ mouse ES cells were electroporated with 10 µg of donor plasmids (*pXL-T3-Neo-UGm-cHS4X; UGm*) and 2 µg of helper plasmids (*pTriEX-mPB* or *pTriEx-NP-mPB*). Electric pulses were provided by a BTX Electro Square Porator EM830 (Harvard Apparatus, Inc., Holliston, MA, USA; 230 V, 0.77 ms). After electroporation, each cuvette of mouse ES cells was seeded onto a 10-cm plate with a feeder layer. One day after electroporation, sequential drug selection was conducted with 200 µg/ml G418 for 2 weeks. Mouse ES cells were fixed by methanol and stained by 0.5% toluidine blue for 15 min. Colonies were washed twice with PBS and dried at room temperature for 30 min. Transposition efficiency was evaluated by the number of surviving colonies per plate.

To determine the PBase transposition efficiency in human ES cells, H9 cells were cultured with 10 µM of rho-associated kinase (ROCK) inhibitor (Y-27632; Calbiochem, San Diego, CA) 2 to 4 h before electroporation, to prevent apoptosis during electroporation. For each electroporation, 10 µg of donor plasmid (*UGm*) and 10 µg of helper plasmid (*pTriEX-mPB* or *pTriEX-NP-mPB*) were mixed with 10^7^ H9 cells. After electroporation, each cuvette of human ES cells was seeded onto a 10-cm plate with a feeder layer. The cells were cultured with medium containing 10 µM ROCK inhibitor. On day 2 after electroporation, cells were cultured with medium without ROCK inhibitor. On days 3 to 7, cells were cultured with a selection medium containing 50 µg/ml G418. On days 8 to 9, the G418 dosage was increased to 100 µg/ml. On day 10, surviving colonies were stained by 0.5% crystal violet for 15 min. The colonies were washed with PBS three times and dried at room temperature for 30 min.

To determine the PBase transposition efficiency in a human cancer cell line, 6×10^6^ Hela cells were transfected in a 10-cm plate with 10 µg of donor plasmids (*pXL-T3-Neo-UGm-cHS4X; UGm*) and 2 µg of helper plasmids (*pTriEX-mPB* or *pTriEx-NP-mPB*) using a transfection reagent (Maestrogen Inc., Las Vegas, NV, USA). After transfection, drug selection was conducted with 200 µg/ml G418 for 10 days. Hela cells were fixed by methanol, stained by 0.5% crystal violet, and the transposition efficiency was evaluated by the number of surviving colonies per plate.

A similar transposition efficiency assay was performed in HEK293T cells. Because HEK293T cells are intrinsically neomycin-resistant, the *UGm* transposon was replaced by the *pGG134* transposon carrying the hygromycin resistance gene cassette [35]. To compare the transposition efficiency mediated by different amounts of PBase, 5×10^6^ HEK293T cells were transfected with 10 µg of donor plasmids (*pGG134*) and 0.25 to 2 µg of helper plasmids (*pTriEX-mPB* or *pTriEx-NP-mPB*). One day after transfection, one-fifth of the cells were split into another 10-cm plate. After selection by 200 µg/ml hygromycin for 7 days, HEK293T cells were fixed by methanol, stained with 0.5% crystal violet, and the transposition efficiency was evaluated by the number of colonies per plate. In the comparison of transposition efficiency, Student’s *t* test was applied to determine the significance of the difference.

### Polymerase Chain Reaction (PCR) Assays for Integration Site Analysis

**Figure 1:**
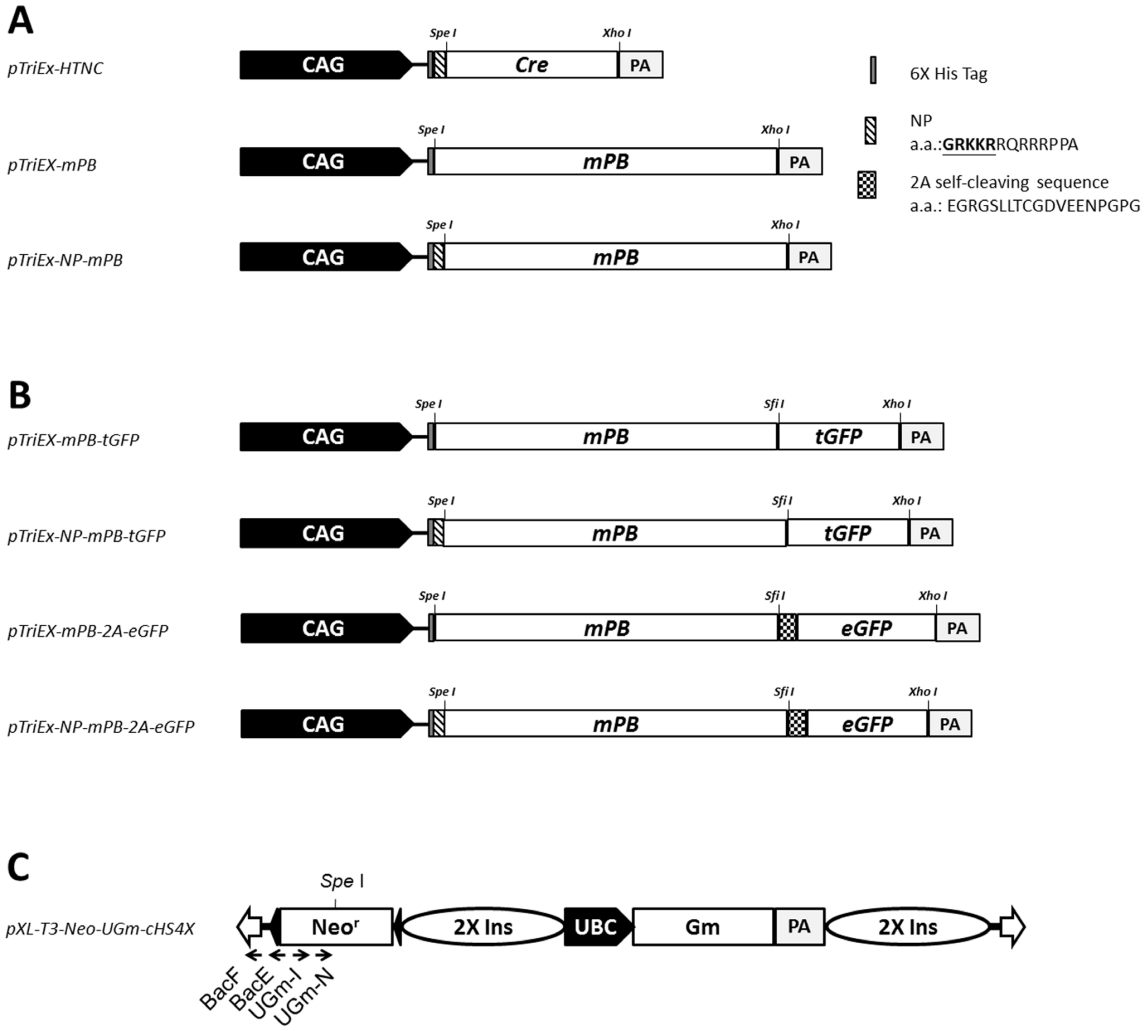
Schematic representation of expression constructs for PBase variants, PBase fusion proteins, and the dual fluorescent *UGm* transposon used in this study. (A) The mPB and NP-mPB coding sequences of mouse codon-optimized PBase were cloned into the *pTriEx-HTNC* plasmid by replacing the *Cre* cassette between *Spe*I and *Xho*I. The mPB and NP-mPB coding sequences were preceded by a hexa-histidine encoding sequence (6× His tag). NP-mPB included an additional nucleolus-predominant (NP) signal peptide. (B) The *pTriEx-mPB-tGFP* and *pTriEx-NP-mPB-tGFP* constructs expressed tGFP-fused PBases. The tGFP moiety allowed real-time imaging of the PBase variant subcellular distributions. The *pTriEx-mPB-2A-eGFP* and *pTriEx-NP-mPB-2A-eGFP* plasmids expressed PBase variants, which were linked to eGFP by the self-cleaving 2A peptide. All protein-encoding cassettes were transcriptionally regulated by a hybrid promoter composed of the CMV immediate early enhancer fused to the chicken β-actin promoter (CAG), and followed by a polyadenylation signal sequence (pA). (C) Flanking the 5′ and 3′ inverted terminal repeats (ITRs) (empty arrows), the dual fluorescent transposon (*pXL-T3-Neo-UGm-cHS4X*; *UGm*) carried a human ubiquitin C (UBC) promoter-driven *H2B-eGFP-2A-mCherry-GPI* (Gm) transgene that labeled the transposed cells with a characteristic chromatin EGFP and membrane mCherry dual fluorescence. Additional abbreviations: Neo^r^, a neomycin phosphotransferase expression cassette providing resistance to G418 selection; 2× Ins, two copies of the insulator sequence from chicken β-globin.

Mouse ES cells were subjected to inverse PCR, as described previously [16]. Briefly, *Sp*el was used to digest 1.5 µg of genomic DNA and then was inactivated. UGm primers (UGm-I and UGm-N) and Bac primers (BacE and BacF) were used to perform nested PCR reactions on self-ligated *Sp*el-restricted genomic DNA (Figure 1C). Amplified DNA fragments were fractionated and purified for direct sequencing with the primer BacF. The following primer sequences were used: UGm-I: 5′-AATTCCTGCA GCCCAATTCC GATC-3′; UGm-N: 5′-TCTGAAGAGG AGTTTACGTC CAGC-3′; BacE: 5′-AGTGACACTT ACCGCATTGA CAAG-3′; and BacF: 5′-TCCTAAATGC ACAGCGACGG ATTC-3′.

The chromosomal location of the transposon was analyzed by the mouse BLAT Search program provided by the University of California, Santa Cruz (http://genome.ucsc.edu; GRCm38/mm10). Insertion sites of transposons were mapped to the mouse genome. These sites fulfilled the following three criteria: they (i) contained the ITR sequence from the nested primer to the end of the 5′ ITR, (ii) showed ≥96% identity to the genomic sequence over the high-quality sequence region, and (iii) had the highest alignment score. In this study, the gene region was specified as the genomic location containing the coding region and the extended region. The coding region was defined as the coordinates from the first base of exon 1 to the last base of the final exon of the longest isoform, to avoid ambiguous counts. The extended region included the sequences extending 5-kb upstream and downstream of a gene. The non-gene region was the genome interval between gene regions. All of the bioinformatics data of exons, introns, and non-gene regions were obtained from the UCSC Genome Browser website.

### Protein Extraction and Western Blot Analysis

HEK293T cells transfected with *pTriEx-mPB* and *pTriEx-NP-mPB* were washed twice with ice-cold PBS and lysed in lysis buffer (25 mM Tris, pH 7.5, 150 mM NaCl, 1% NP-40, 1 mM EDTA, 1 mM Na_3_VO_4_, 1 mM NaF, 10 µg/ml aprotinin, and 50 µM ρ-APMSF). The lysates were centrifuged, and the protein extracts were separated on 8% SDS-PAGE and transferred to Immobilon transfer membranes (PVDF) (Millipore, Bedford, MA, USA). The membranes were washed, incubated in TBST containing 5% skim milk for 1 h, and treated overnight with an anti-His tag (1∶2000; GeneTex, Inc., Irvine, CA, USA), anti-GFP (1∶5000; GeneTex, Inc.), or anti-α-tubulin antibody (1∶5000; Abcam plc, Cambridge, UK) at 4°C. A horseradish peroxidase-conjugated secondary antibody (1∶5000; GeneTex, Inc.) was applied to allow color reaction for detection. For nucleo-cytoplasmic separation experiments, cytoplasmic and nuclear protein fractions were extracted from transfected HEK293T cells with the ProteoJET cytoplasmic and nuclear protein extraction kit (Thermo Fisher Scientific Inc.).

### Protein Stability Assay

To test the protein stability of PBase variants, HEK293T cells were transfected with PBase-expressing plasmids (*pTriEX-mPB-2A-eGFP* and *pTriEX-NP-mPB-2A-eGFP*) using the Maestrofectin transfection reagent (Maestrogen Inc.). Transfected cells were harvested 2 days later and stained with allophycocyanin (APC)-conjugated anti-His tag antibody (1∶500, GeneTex, Inc.). The specimens were strained through BD Falcon cell strainers and kept on ice before acquisition. Flow cytometry analysis was performed with a FACS AriaII (BD Bioscience Inc., San Jose, CA, USA) facilitated by FlowJo software (Tree Star, Inc., Ashland, OR, USA). In all samples, >10,000 cellular events from the defined cell cluster were analyzed per tube.

### Cytotoxicity Assay

To analyze the potential cytotoxicity caused by mPB and NP-mPB, HEK293T cells were transfected with different amounts of PBase-expressing vectors, and cell mortality was measured by a (3-(4,5-dimethylthiazol-2-yl)-5-(3-carboxymethoxyphenyl)-2-(4-sulfophenyl)-2H-tetrazolium) (MTS) assay. Approximately 5×10^3^ HEK293T cells were seeded in each well of a 96-well plate on the day before transfection. Maestrofectin transfection reagent (0.4 µl; Maestrogen Inc.) was mixed with different amounts of PBase-expressing vectors (0–300 ng) in 10 µl of serum-free DMEM and left at room temperature for 20 min. Liposome mixtures were applied to the wells and transfected overnight.

After 3 days, the cells were subjected to a CellTiter 96 AQueous Non-Radioactive Cell Proliferation Assay (Promega), in accordance with the manufacturer’s instructions. The relative cell viability was estimated by absorbance at 490 nm, as measured by a MULTISCAN Microplate Absorbance Reader (Thermo Fisher Scientific) directly from 96-well culture plates and computed with Microsoft Excel software (Microsoft, Redmond, WA, USA). All values are presented as the mean ± standard error of three independent experiments. One-way analysis of variance (ANOVA) was applied to compare the cell viabilities after different PBase vectors were transfected in the same amount. Bonferroni adjustment was done for the *post hoc* multiple comparisons to avoid inflated type I error.

### Immunocytochemistry and Fluorescent Confocal Microscopy

Hela cell explants were fixed with 4% paraformaldehyde in PBS for 20 min at room temperature, washed twice with PBS, permeabilized for 20 min using 0.3% Triton X-100/PBS, and blocked for 30 min in blocking buffer (10% normal goat serum and 2% bovine serum albumin/PBS). Hela cell explants were incubated with anti-cyclin T1 (1∶200; Santa Cruz Technology, Santa Cruz, CA, USA) and anti-fibrillarin (1∶200; Abcam plc) antibodies, diluted in blocking buffer at 4°C overnight, and incubated for 1 h at room temperature with Alexa Fluor 568- and Alexa Fluor 647-conjugated secondary antibodies (Invitrogen). Fluorescence microscopic images were captured and processed using a Leica TCS SP5 confocal spectral microscope imaging system (Leica Microsystems GmbH, Heidelberger, Germany).

## Results

### Construction of the NP-mPB–expressing Vector

To direct the mPB toward the nucleolar compartment, we added an NP signal peptide sequence (GRKKR) from the HIV-1 TAT protein to the N-terminus of mPB. The resulting NP-mPB fusion was constructed with a *pTriEX-HTNC*-derived plasmid [60]. The *pTriEx-NP-mPB* construct was composed of a CAG promoter-driven mPB variant with a preceding (His)_6_ tag and an NP signal peptide (Figure 1A, bottom). In the control *pTriEx-mPB* plasmid, the coding sequences for the promoter and mPB were kept the same, except that the NP peptide-encoding sequence was removed (Figure 1A, middle).

To elucidate whether the NP peptide directs the transposase to nucleoli as predicted, constructs encoding mPB and NP-mPB fused in-frame with tGFP were made, and the subcellular localization of the transposase was traced under confocal microscopy (Figure 1B, top two constructs). We also made constructs that linked either mPB or NP-mPB to an eGFP moiety using a self-cleaving T2A peptide, and compared whether the addition of the NP peptide altered the transposase protein level through the translation or degradation of the peptide (Figure 1B, bottom two constructs). The transposition efficiency mediated by each transposase variant was tested with a previously described, approximately 12-kB *piggyBac* transposon, *pXL-T3-Neo-UGm-cHS4X* (*UGm*), which carried both dual fluorescence and neomycin-resistance expression cassettes. By using this transposon, PBase-mediated transposition events could be selected with G418 and verified by the dual fluorescence appearance of the surviving colonies (Figure 1C, Figure S1) [16].

### NP-mPB Mediates Elevated Transposition Efficiency in Mouse and Human Cells

To evaluate whether the PBase-mediated transposition efficiency was affected by the additional NP peptide, either *pTriEx-mPB* or *pTriEx-NP-mPB* was co-electroporated with the *pXL-T3-Neo-UGm-cHS4X* plasmid into either mouse or human ES cells. The number of colonies surviving G418 selection was used to measure transposition efficiency mediated by different PBase variants. The expression of dual fluorescence under fluorescent microscopy was used to reconfirm the transposon integration events (Figure S1).

**Figure 2:**
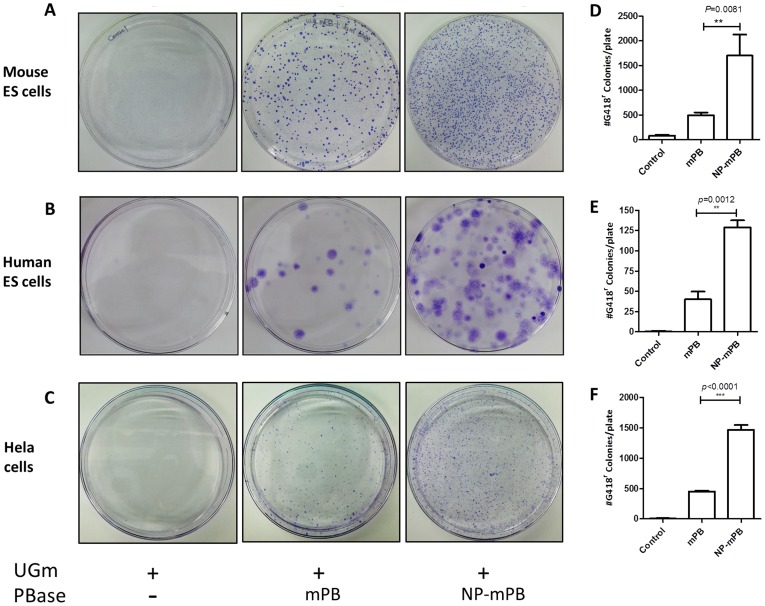
The NP-mPB showed a 3- to 4-fold increase in transposon integration efficiency. The *UGm* transposon, alone or mixed with the mPB or NP-mPB expression vector, was co-electroporated into mouse or human embryonic stem (ES) cells and then selected for G418 resistance. Surviving colonies were stained by crystal violet and counted. There were more colonies in the NP-mPB group than in the mPB group in mouse ES (A), human ES (B), and Hela cells (C). (D–F) Transposition events were quantified by counting the colonies on culture plates. The transposition efficiency mediated by NP-mPB was increased 3- to 4-fold in mouse ES cells (D), 3-fold in human ES cells (E), and approximately 3-fold in Hela cells (F). n  = 3 for each condition; bars indicate mean ± standard error; ** *P*<0.01, *** *P*<0.001.

**Figure 3:**
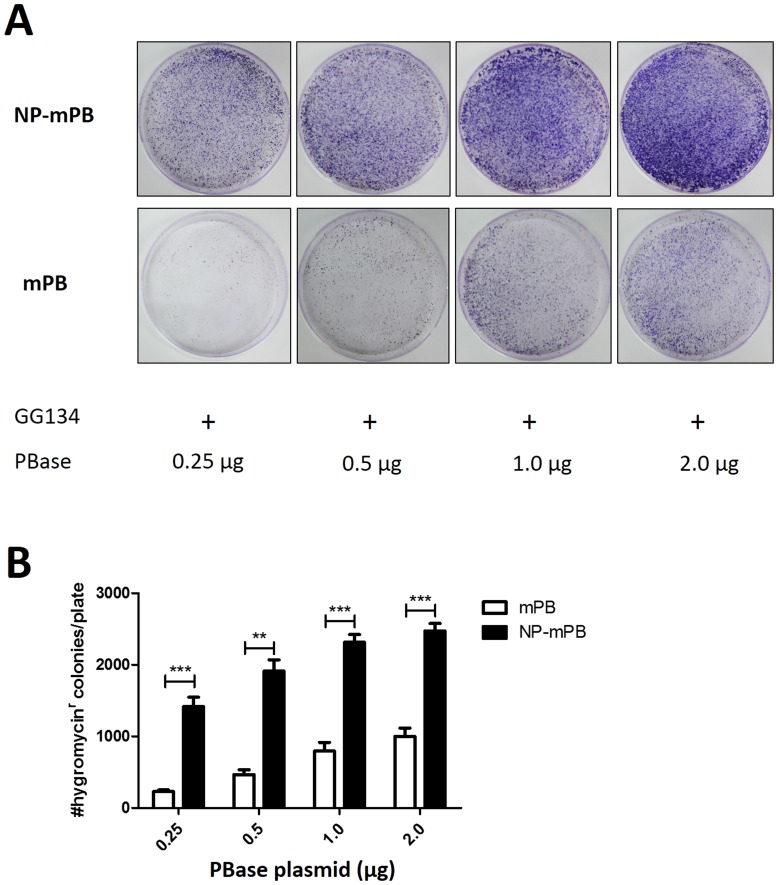
Evaluation of the transposition efficiency mediated by the gradient concentrations of PBase. (A) The NP-mPB group demonstrated more colonies compared to the mPB group in HEK293T cells transfected with varying amounts of plasmid DNA. (B) Colony numbers were quantified, revealing a 2.5- to 6-fold increase in transposon integration efficiency for NP-mPB. The transposition efficiency was higher when more plasmid was transfected for both groups. No overproduction inhibition was noted. n  = 3 for each condition; bars indicate mean ± standard error; ** *P*<0.01, *** *P*<0.001.

In mouse ES cells, the transposition efficiency mediated by NP-mPB was about 3.5-fold that by mPB (Figure 2A, 2D). In human ES cells, the transposition efficiency mediated by NP-mPB was 3.2-fold that by mPB (Figure 2B, 2E). In Hela cells, the transposition efficiency mediated by NP-mPB was about 3.2-fold that by mPB (Figure 2C, 2F). Clearly, there was a significant, >3-fold increase in transposition efficiency after the NP peptide was added to mPB. In HEK293T cells, the transposition efficiency was studied by transfection with gradient concentrations of PBase. The transposition efficiency of NP-mPB was 2.5 to 6 times that of mPB (Figure 3).

For mPB and NP-mPB, no obvious overexpression inhibition was found within the range of PBase-expressing vector amounts in our study because the transposition efficiency was higher when more PBase was transfected. The differences of transposition efficiency between the NP-mPB and mPB groups were smaller when larger amounts of plasmid DNA were transfected.

### NP-mPB does not Mediate Transposon Insertional Preference Towards NORs

To examine whether addition of the NP peptide drove the transposon insertional preference towards NORs, we sequenced the NP-mPB–mediated insertion sites and located them on the mouse genome sequence. Single mouse ES cell-derived colonies were picked for inverse PCR to identify the transposon insertion sites. Twelve clones were expanded, PCR-amplified, and sequenced (Table 1).

**Table 1:** Insertion site analysis of NP-mPB assisted transposition events.

	Donor Sequence	Flanking Sequence	Location	Gene
1	TTTCTAGGG**TTAA**AAAGCATGTAAAAAGCATGAGAAA		Chr3∶10,381,388-9	*Chmp4c*
2	TTTCTAGGG**TTAA**aTATCTGTTCAGTTTTATAGGAAG		Chr4∶124,049,072-3	Non-gene
3	TTTCTAGGG**TTAA**ACAAGCATAAACTCGACAATGACA		Chr4∶134,111,453-4	*Ubxn11*
4	TTTCTAGGG**TTAA**ACAGGGTTGTGCAACTGTTTTAAA		Chr4∶138,725,589-90	*Pla2g2c*
5	TTTCTAGGG**TTAA**AGGCtaTCCTCTGTCCCTGGTCTC		Chr5∶125,504,911-2	*Aacs*
6	TTTCTAGGG**TTAA**CTGCTGAACCATCTCTTCAGCTCC		Chr9∶113,277,688-9	Non-gene
7	TTTCTAGGG**TTAA**AAGCCCACTCATCTACGGATGAGA		Chr10∶3,242,756-7	Non-gene
8	TTTCTAGGG**TTAA**TTAGTTCCCTGAAGGTTTACCAAA		Chr12∶6,656,096-7	Non-gene
9	TTTCTAGGG**TTAA**TCAAGAAAATGTACAACAGGTTTG		Chr13∶12,534,269-70	Non-gene
10	TTTCTAGGG**TTAA**TTCTGTTCTCCTGTGAGTGGTTGT		Chr14∶26,988,052-3	Non-gene
11	TTTCTAGGG**TTAA**TCAAGGCTGATGAGGTCTTACAAC		Chr16∶18,202,292-3	Non-gene
12	TTTCTAGGG**tTAA**aTaTGCTTAGATGCCCCAGTGGAA		Chr17∶83,850,723-4	*Haao*

The lowercase letters indicate the unmatched nucleotides in the flanking sequence alignment.

The mouse genome BLAT search program revealed that the 12 insertion sites were distributed through 10 of the 20 chromosomes in the mouse genome (*i.e.*, chromosomes 3–5, 9, 10, 12–14, 16, and 17). The distribution was relatively random, except for three sites in chromosome 4. Five of the 12 insertion sites were in the gene region; the other 7 sites were in the non-gene region. None of the insertion sites were in the NOR-defined rRNA gene clusters.

### The NP-mPB Protein Level does not Explain the Increased Transposition in Mammalian Cells

**Figure 4:**
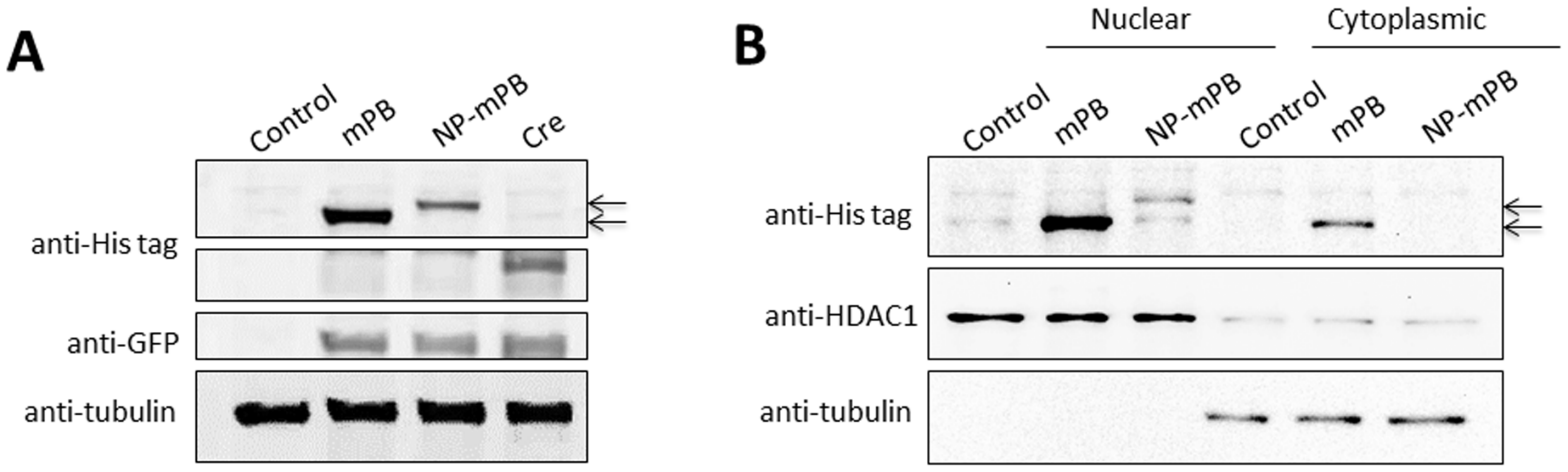
The mPB and NP-mPB protein expression profiles in HEK293T cells transfected with *pTriEx-mPB* and *pTriEx-NP-mPB* plasmids. (A) At 48 h after transfection, the transposase protein level in the *pTriEx-mPB* group was higher than that in the *pTriEx-NP-mPB* group. *UGm* plasmid was cotransfected as a control for transfection efficiency (anti-GFP). *pTriEX-HTNC* plasmid was transfected as a positive control for anti-His tag antibody (Cre). (B) Western blot analysis of nucleo-cytoplasmic separated lysates revealed that the mPB protein was distributed preferentially in the nucleus and the cytoplasm, whereas almost all NP-mPB protein was localized in the nucleus.

To decipher the cause of the increased transposition by NP-mPB in mammalian cells, the PBase protein expression profile was surveyed. One hypothesis to explain the elevated transposition efficiency is that there was an increased PBase protein accumulation in the NP-mPB–transfected cells that resulted from either elevated expression or decreased degradation of PBase. To test this hypothesis, HEK293T cells were transfected with *pTriEx-mPB* or *pTriEx-NP-mPB* by a liposome-based protocol. The transfection efficiency was >90%, as revealed by fluorescent microscopy of cotransfected *UGm* plasmid expression. We confirmed the specificity of the anti-His tag antibody to identify mPB and NP-mPB and their molecular weights on SDS-PAGE by an immunoprecipitation experiment followed by a LC-MS/MS assay (Figure S2). However, the total transposase protein of NP-mPB was less than that of mPB (Figure 4A). Therefore, the total amount of transposase expression did not account for the increased transposition efficiency of NP-mPB.

Another hypothesis is that NP-mPB was more concentrated in the subcellular compartment(s) where transposition occurred. Because adding NP signal peptide theoretically leads to nuclear accumulation, Western blot was performed after nucleo-cytoplasmic separation to determine the relative amount of protein in the nucleus. For both NP-mPB and mPB, most of the transposase proteins were in the nucleus. Compared to mPB, the absolute amount of NP-mPB protein in the nucleus was comparatively less. Therefore, the elevated transposition efficiency mediated by NP-mPB was not due to an increased nuclear accumulation of PBase protein.

### The Protein Stability of NP-mPB is Decreased Compared to that of mPB

Although the *cis*-transcriptional regulatory elements were identical on *pTriEX-mPB* and *pTriEX-NP-mPB*, the *pTriEX-NP-mPB*–transfected cells showed less PBase protein on Western blot analysis. To examine whether this finding was due to a difference in the translation and/or degradation of PBase protein, we created plasmids expressing the mPB-2A-eGFP and NP-mPB-2A-eGFP fusion proteins. Then, the PBase protein stability was determined by flow cytometry of Hela cells transfected with either *pTriEx-mPB-2A-eGFP* or *pTriEx-NP-mPB-2A-eGFP*. The presence of transposase (mPB and NP-mPB) was evaluated by using an APC-conjugated anti-His tag antibody. Because the eGFP moiety was first translated in the PBase fusion proteins, but then separated from PBase by self-cleavage of the 2A peptide, the eGFP intensity provided an estimate of the mPB and NP-mPB production independent of subsequent degradation events.

**Figure 5:**
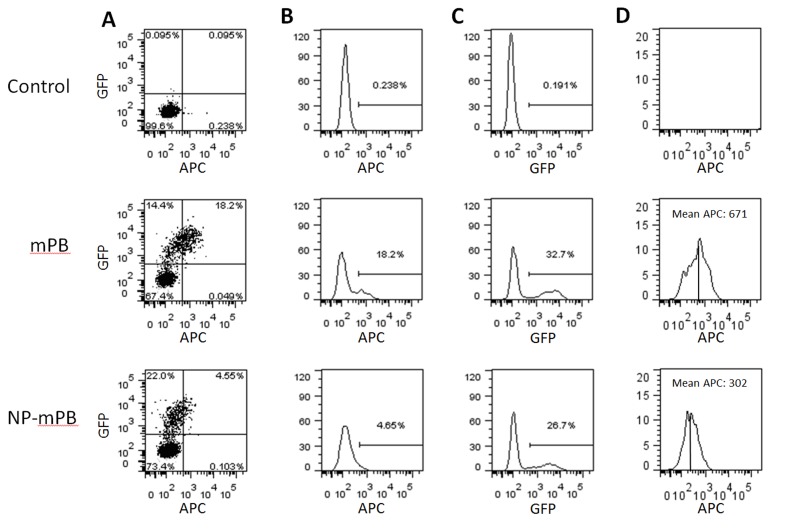
Stability analysis of PBase protein, facilitated by flow cytometry. Hela cells were transfected with *pTriEx-mPB-2A-eGFP* and *pTriEx-NP-mPB-2A-eGFP* plasmids. (A) APC fluorescence via anti-His tag antibody staining indicated the presence of mPB or NP-mPB. Because the eGFP moiety was cotranslated with PBase variants, the GFP fluorescence intensity was used as a reference for the mPB and NP-mPB translation levels of successfully transfected cells. (B and C) Y-axis represents accumulated cell counts. Although the NP-mPB group had a much smaller percentage of cells that were APC+ (4.65%) than the mPB group (18.2%), the percentages of GFP+ cells were similar (26.7% vs. 32.7%). (D) The APC fluorescence intensity in GFP+ populations represented the protein stability. The ratio of mean APC fluorescence (a.u.) of NP-mPB to mPB was 302 to 671.

The two transfected populations only showed a slight difference in eGFP intensity distribution on flow cytometry, indicating that similar amounts of mPB and NP-mPB were translated (Figure 5A, 5C). However, compared to cells from the *pTriEx-mPB* transfection experiment, close to 4-fold fewer cells from the *pTriEX-NP-mPB* transfection experiment displayed an APC fluorescent intensity that exceeded the threshold (Figure 5A, 5B). Furthermore, when we only considered the APC fluorescent intensity of cells from the eGFP+ population from both experiments, the mean APC fluorescent intensities were 671 arbitrary units (a.u.) for *pTriEX-mPB* and 302 a.u. for *pTriEX-NP-mPB* transfectants. These findings might imply an increased protein degradation of NP-mPB (Figure 5D).

### NP-mPB Overexpression Causes Little Cytotoxicity

**Figure 6:**
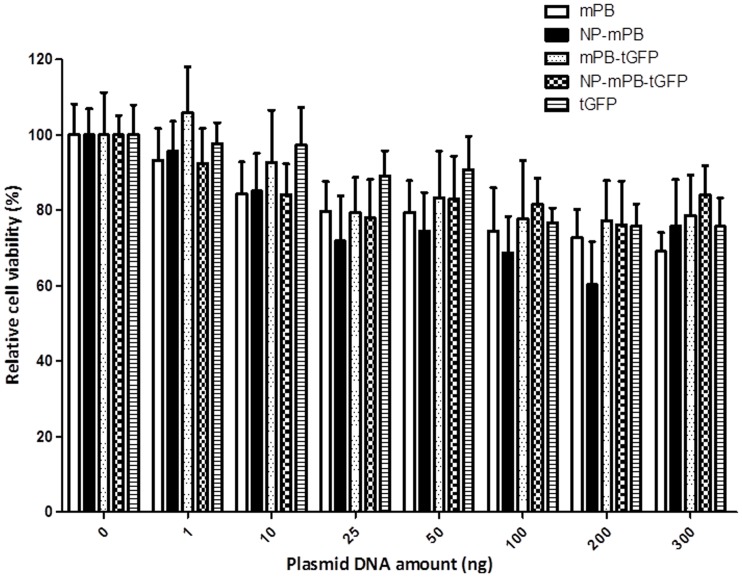
NP-mPB expression has little effect on cell viability. To probe the potential cytotoxicity of mPB and NP-mPB expression, HEK293T cells were transfected with different amounts of the mPB, NP-mPB, mPB-tGFP, and NP-mPB-tGFP expression vectors. The relative cell viability was evaluated by the MTS assay. There was no significant difference among the different protein-expressing vectors. n  = 3 for each condition; bars indicate mean ± standard error; *P*>0.05.

To rule out the possibility that NP-mPB causes cytotoxicity, HEK293T cells were transfected with *pTriEx-mPB*, *pTriEx-NP-mPB*, *pTriEx-mPB-tGFP*, or *pTriEx-NP-mPB-tGFP* separately (Figure 6). Cells transfected with a tGFP expression vector were used as a negative control. In transfection experiments using different amounts of PBase-expressing vectors, the survival rates of the control, mPB, and NP-mPB groups were not significantly different. Thus, we found no evidence to suggest that NP-mPB overexpression caused an increase in cytotoxicity.

### The NP Signal Directs NP-mPB to a Predominantly Nucleolar Distribution

**Figure 7:**
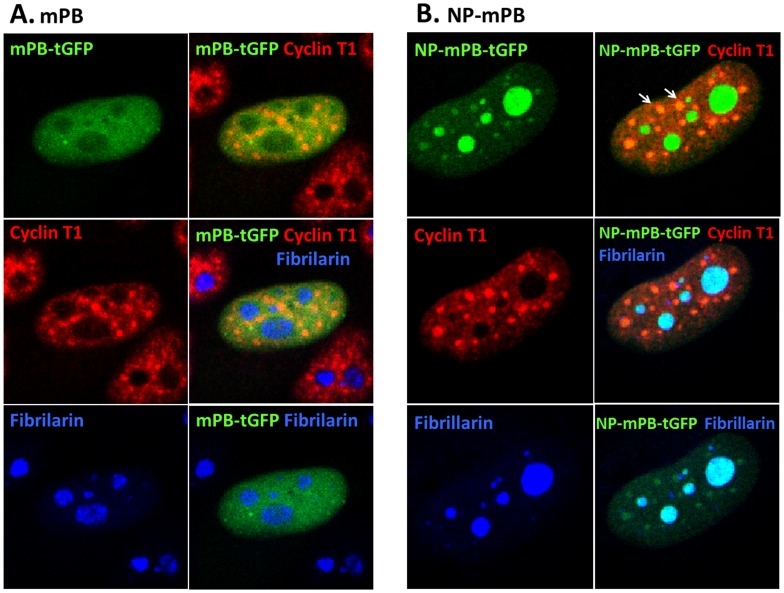
Subcellular localization of mPB and NP-mPB. Hela cells were transfected with mPB-tGFP or NP-mPB-tGFP expression vectors and immunostained with an anti-cyclin T1 antibody (red) and an anti-fibrillarin antibody (blue). (A) Fluorescence microscopy revealed that mPB was localized in the nucleus, but largely outside the nucleoli, as indicated by the fibrillarin. The cyclin T1 signal was dotted and spared the nucleus. (B) In contrast, NP-mPB was localized in the nucleus, with a strong staining in the nucleoli. There was a dotted pattern of NP-mPB in the nucleus beside the nucleoli. Some of the dots were overlapped with the cyclin T1 signal.

For transposase to mediate transposition events, it should localize to somewhere near its substrate (genomic DNA in the nucleus). Therefore, it was reasonable to assume that a more efficient delivery of PBase to its target site could increase the transposition rate. NP-mPB theoretically has a preference to locate in the nucleolus. To define the distribution of mPB and NP-mPB in the subcellular compartments, Hela cells were transfected with either *pTriEx-mPB-tGFP* or *pTriEx-NP-mPB-tGFP*, and then immunostained with anti-cyclin T1 and anti-fibrillarin antibodies. Fluorescence microscopy revealed that both mPB and NP-mPB proteins located mainly in the nucleus. However, whereas NP-mPB was obviously concentrated in the nucleoli and other dots overlapping with cyclin T1 signals (Figure 7), mPB was evenly distributed in the nucleus, relatively sparing the nucleoli.

## Discussion

The function of mPB has been demonstrated to remain intact when mPB is fused with different proteins, such as with the Gal4 or zinc-finger DBD, to create a targeted transposition system [53], [54]. In this study, PBase was fused with the PTD of the HIV-1 TAT protein (N’-GRKKRRQRRRPPA-C’). The PTD traverses the cell membrane to promote cellular uptake of the fusion protein [66], [67], and may enhance membrane penetration directly or by endocytosis [68], [69]. Additionally, the fusion cargo influences the permeabilization mechanism [70], and the delivery protocol impacts the transmembrane efficiency and final subcellular localization of the fusion protein. Studies have demonstrated that liposome-based transient transfection of TAT-GFP in mammalian cells permits nuclear localization of the fusion protein [71], [72]. It has also been shown that the PTD of TAT protein contains nucleolar localization signal [62], [63] [73]–[76].

Our initial goal was to create a modified mPB that would mediate transposition in the NORs, where gene disruption would typically not be harmful. Eleven amino acids of the PTD peptide from TAT sequences were added to the N-terminal region of mPB as an NP signal peptide, to target PBase to the proximity of NORs in a transient transfection system. However, analysis of the insertion site did not reveal a transposition preference for NORs. The transposition efficiency mediated by NP-mPB was increased by 3- to 4-fold, with about 40% of integrations occurring in the gene region.

Although modification of transposase by ligation with a signal peptide or protein can theoretically change the transposition preference, it is not always altered as expected. One example includes the newly developed excision competent/integration defective PBases. When these PBases were fused with a *ROSA26*-binding ZNF to restore the integration ability, transposon insertion into *ROSA26* target sites was not obviously increased [77]. Likewise, in this study, NP-mPB was not successful at targeting a specific gene region (*i.e.*, NORs). However, the significant increase of transposition events mediated by NP-mPB was an advantageous finding.

To investigate the mechanism of increased transposition efficiency, we assessed the PBase protein expression profile. Compared to mPB, the NP-mPB was associated with a higher integration rate for a lower amount of protein present. The Western blot analysis of the nucleo-cytoplasmically separated samples also indicated that NP-mPB was less enriched in the nucleus compared to mPB when equal amounts of the plasmids were transfected. To rule out the possibility that the reduced NP-mPB production was due to toxicity with plasmid transfection, a cell survival assay was performed. The data did not demonstrate a significant cytotoxicity difference between mPB and NP-mPB at various concentrations. However, the protein stability assay suggested that NP-mPB is less stable than mPB and, therefore, may have a higher degradation rate, which could explain its lower amount of protein. Nevertheless, from the transposition efficiency tests using different amounts of PBase-expressing vectors in HEK293T cells, overexpression inhibition is unlikely to explain the observed transposition efficiency differences between the PBase species in our experiment.

It is worth probing the mechanism for the higher transposition efficiency but lower protein presentation of NP-mPB. One possible explanation is the targeted delivery ability of the fusion protein. The TAT PTD permits the fused protein to localize to the nucleus, specifically in the subnuclear compartments [76]. Two factors that are closely associated with TAT in the nucleus are fibrillarin and cyclin T1. TAT was reported to associate with the fibrillarin-U3 snoRNA complex in *Drosophila* nurse cell nucleoli [74]. Although fibrillarin colocalized with TAT in the nucleoli in another study, no direct protein-protein interaction was found by fluorescence resonant energy transfer (FRET). Cyclin T1 is a transcriptional elongation factor that interacts with TAT and the transactivating response element to mediate activation of some lentiviral promoters [78]–[81]. Visualization of FRET between TAT and cyclin T1 proved the direct protein-protein interaction [76].

Because of the many advantages of the *piggyBac* system for mammalian functional genomics and regenerative medicine research, substantial effort has been put forward to improve the expression level and enzyme efficiency of PBase. Transcriptional abundance can be ensured through the use of *cis*-regulatory elements, such as strong promoters mediating high-level transcription and effective polyadenylation signals to increase mRNA stability. Studies have sought to optimize codon usage for translation in mammals [50]. Using error-prone PCR and high-throughput screening, researchers generated a hyperactive mPB, named hyPBase [82], which featured a 9-fold increase in transposon integration and a 17-fold increase in excision compared to the original mPB. Fusion of an NP signal peptide to mPB could contribute to an increase in transposition efficiency.

In this study, we demonstrated that NP-mPB was restricted to the nucleus and associated with fibrillarin and cyclin T1. Unexpectedly, the transposon insertion sites were relatively random, except for a tendency to be in the gene region, which was similar to a previous report for mPB [44]. There was no obvious preference for the (fibrillarin-associated) NORs. Therefore, the purpose of constructing NP-mPB to target the transposon to the NORs and to prevent essential gene disruption was not successful. However, the resulting NP-mPB exhibited improved transposition efficiency and relatively random transposon integration.

The PBase was revised in several ways to improve its transposition efficiency. The developed NP-mPB exhibits enhanced efficiency without increased toxicity, which is desirable for transgenesis, insertional mutagenesis, and gene therapy. In addition, the NP-mPB does not alter the random insertional preference of the transposon and, therefore, maintains the advantage for establishing a mutagenesis library. In the future, linkage of the NP signal peptide to other modified transposases could possibly be used to increase the efficiency further.

## Supporting Information

Figure S1 **Confirmation of the** ***UGm*** **transposon integration by the presence of dual fluorescence under fluorescent microscopy.** (A) Bright-field and (B) fluorescence microscopy images of a *UGm*-transposed, G418-resistant, developing mouse ES cell colony. (C) Confocal microscopy of a human ES cell colony that survived G418 selection.
(TIF)

Figure S2 **Identification of protein by LC-MS/MS with protein database search.** (A) Coomassie brilliant blue-stained SDS-PAGE results of an immunoprecipitation experiment. An anti-His tag antibody was used to probe HEK 293T cells expressing mPB, NP-mPB, and Cre. Circled bands were cut and used for an LC-MS/MS assay to confirm the identities. (B) The LC-MS/MS results were analyzed by a Mascot probability-based scoring system. For the bands from the mPB and NP-mPB groups, individual ion scores >60, which indicated identity or extensive homology (*P*<0.05), were found in the amino acid sequences of the *piggyBac* transposase from *Trichoplusia ni* (text in red).
(TIF)
