# Supplementary figures and images for: A Nucleolus-Predominant piggyBac Transposase, NP-mPB, Mediates Elevated Transposition Efficiency in Mammalian Cells
Source: PLoS One. 2014 Feb 24;9(2):e89396. doi: 10.1371/journal.pone.0089396 (PMC3933532; doi:10.1371/journal.pone.0089396)

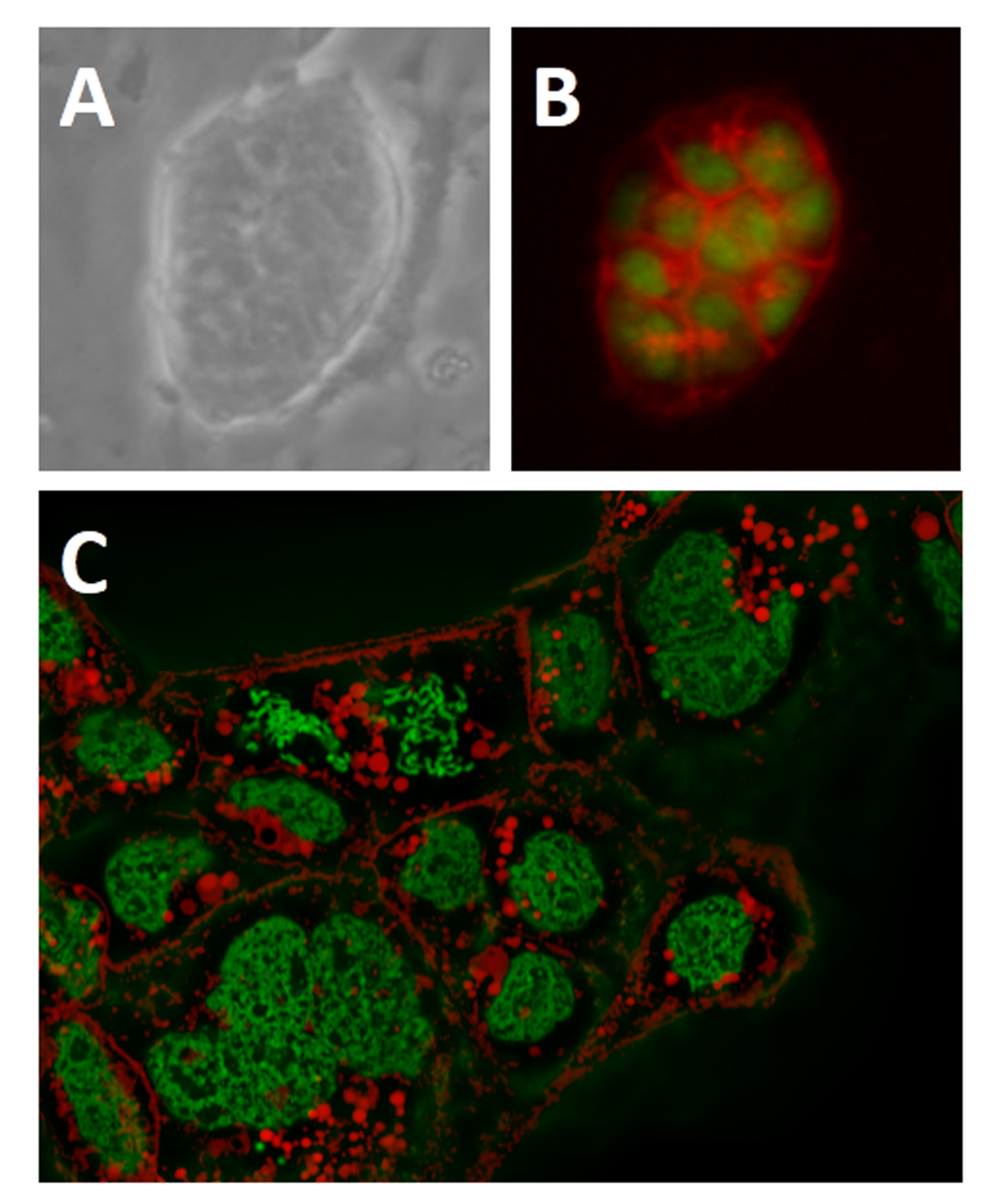

Supplement: Figure S1 — Confirmation of the UGm transposon integration by the presence of dual fluorescence under fluorescent microscopy. (A) Bright-field and (B) fluorescence microscopy images of a UGm-transposed, G418-resistant, developing mouse ES cell colony. (C) Confocal microscopy of a human ES cell colony that survived G418 selection. (TIF) [file pone.0089396.s001.tif]

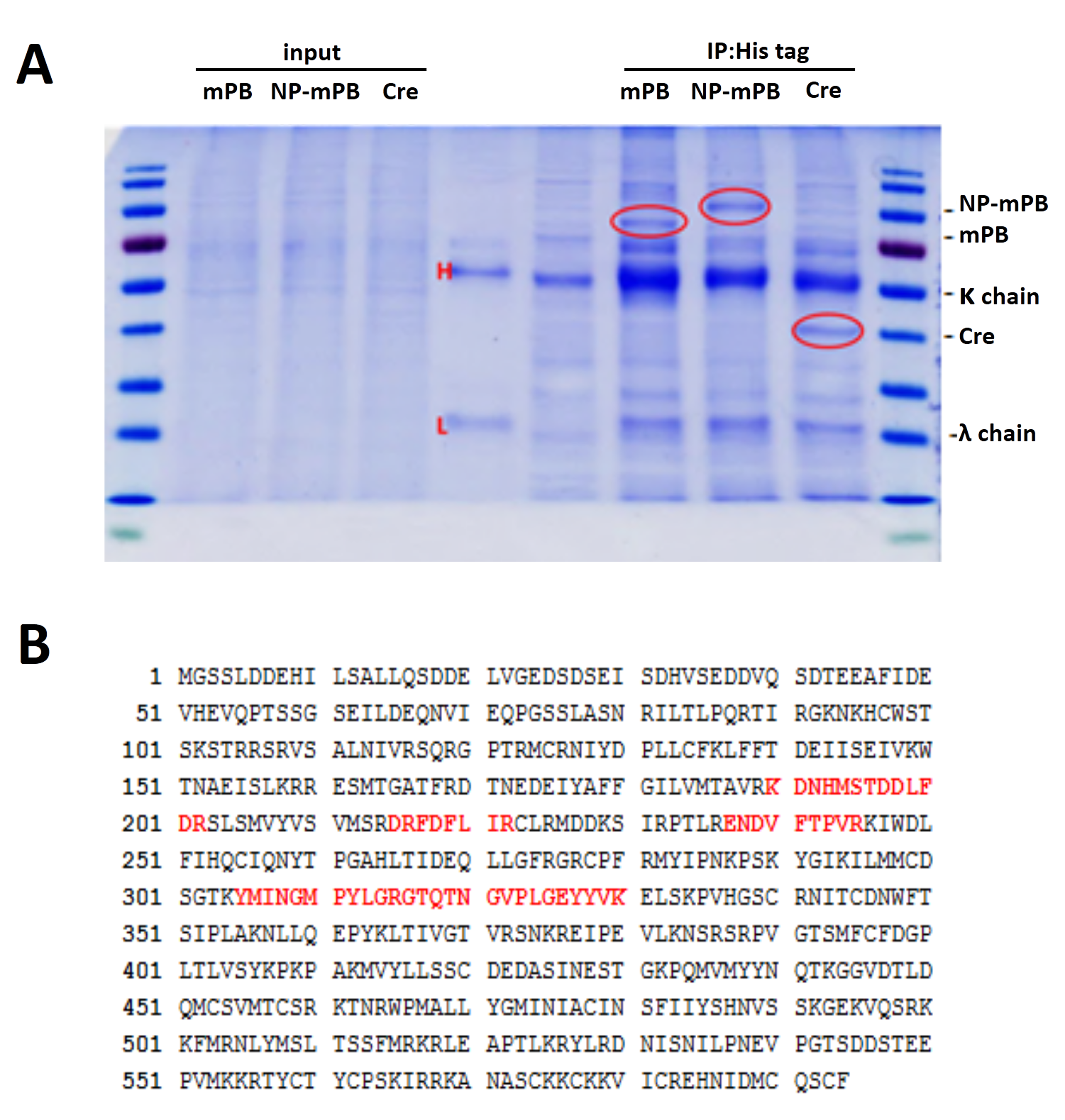

Supplement: Figure S2 — Identification of protein by LC-MS/MS with protein database search. (A) Coomassie brilliant blue-stained SDS-PAGE results of an immunoprecipitation experiment. An anti-His tag antibody was used to probe HEK 293T cells expressing mPB, NP-mPB, and Cre. Circled bands were cut and used for an LC-MS/MS assay to confirm the identities. (B) The LC-MS/MS results were analyzed by a Mascot probability-based scoring system. For the bands from the mPB and NP-mPB groups, individual ion scores >60, which indicated identity or extensive homology (P<0.05), were found in the amino acid sequences of the piggyBac transposase from Trichoplusia ni (text in red). (TIF) [file pone.0089396.s002.tif]
